# 

**Published:** 1886-12-18

**Authors:** 


The Hospital.

An Institution, Family, and Parochial Journal of Hospitals, Asylums, and all Agencies for the Care of the Sick, Criticism and News.

SATURDAY, DECEMBER 18th, 1886.

**Notice to Correspondents.**

Mr. A. O'D. Bartholerns, Secretary of the Middlesex Hospital, is thanked for his kindness in sending a few notes to assist in making the In- and Out-Patients' Diary accurate.

### THE CHILDREN'S CORNER.

### OUR PASTIMES.

**Children's Quarterly Prize Competition.**

| | |
|---|---|
| Began .. .. .. .. .. | 2nd Oct., 1886 |
| Ends .. .. .. .. .. | 25th Dec., 1886 |

PRIZES.

| | |
|---|---|
| 1st .. .. .. .. .. | Thirty Shillings |
| 2nd .. .. .. .. .. | Twenty ,, |
| 3rd .. .. .. .. .. | Ten ,, |
| 4th .. .. .. .. .. | Five ,, |

will be awarded to the four competitors who shall have sent in, during the thirteen weeks, the largest number of correct answers to our pastimes.

**Twelfth Competition.**

No. 61. Can you tell me what was the longest day which Adam spent in Paradise?

No. 62. Buried towns. "Did the hero mean to attempt it?" "You should never play nap lest you lose." "Twenty reckless men scaled the walls."

No. 63. Who can make a little French sentence out of the following: "Je bis à vore"?

No. 64. Take, if you please, the names of our first parents and those of their three sons, and so arrange them that they will make a sentence.

No. 65. Many years ago a letter came into the hands of the postal authorities, addressed "Al Excellenza Srom Fri Devi." The post-office people being unable to interpret the enigma, sent it to an Orientalist at the British Museum, who discovered that the writer intended it for a distinguished chemist. Who was the chemist?

No. 66. Square Word. The early part of the year; a child's elementary book; a word that expresses a gentle movement of the water; to cheat; a great English admiral; a word meaning vegetables. Read downwards, the letters will spell the same words as if read horizontally.

No. 67. What is hated by none,
Yet brings some to bad ends,
Is what I wish for myself
As well as my friends.

**Results of Tenth Competition.**

No. 49. The two animals which went into the ark provided with the toilet necessaries, were, of course, the fox who took his "brush," and the cock who took his "comb."

No. 50. The answer to this is "Medicine."

No. 51. My little readers did not, I fancy, find it difficult to divine the letters of the word "Tobacco" from the geometrical clues I gave.

No. 52. The following are the lights of the acrostic:

J utlan D

E ch O

N arcoti C

N ea T

E lch O

R eindee R

which give us Dr. Jenner, who discovered vaccination.

No. 53. This is a well-known riddle. The sentence can be made to rhyme by "spelling out" the last word thus:

There was an old woman, and she
Was as deaf as a P.O.S.T.

No. 54. *Myself* in a very short word is "I," my second, a *puppet*, gives us "doll," and the whole, of course, "idol."

The competition still goes merrily along, and those of my little friends who are beaten, evidently do not intend to be badly beaten. This week there is quite a little army of "all rights," and the "three right," and "two right," are altogether absent from the list. Remember, my little friends, only one more competition remains after this, to decide the prize-winners of the first quarterly pastime.

All right—Mikado, Florrie, Jimmy, Alsie, Tina, Retsibsi, T. K.

Five right—True Blue, Filomel.

Four right—Little Mary, Bismarck.

Answers, having the actual name and address (to which a pseudonym may be added for publication), must have The Children's" Coupon attached (see advertisement pages), and be addressed to the "PUZZLE EDITOR," THE HOSPITAL, Norfolk House, Norfolk Street, Strand, W.C., not later than Thursday, 23rd December. Results will appear in our issue of the 1st January, 1887.

### "THE HOSPITAL" CHARITY BOX.

OUR "Charity Box" is intended to afford a little practical help to the hospitals. Each week we propose a word for "anatomical dissection," and the first 150 competitors who extract the largest number of words from it receive the following prizes:—

| | |
|---|---|
| 1st .. .. .. .. | One Guinea. |
| 2nd .. .. .. .. | Half a Guinea. |
| 3rd .. .. .. .. | Five Shillings. |

The above Prizes will be repeated for every additional 150 competitors who may enter each week

TWELFTH COMPETITION.

Compose the largest number of words you can from "PATIENT."

*Observe:* Proper names, abbreviations, foreign, Latin, and obsolete words are barred.

Repetitions of similarly spelt words and of letters (unless they repeat in the word) are disallowed.

Plurals allowed, but not the possessive in 's or s'.

Prefixes and affixes do not count independently of words.

Write only on one side of the paper, and total your words.

Append your real name and address, adding whether Mr., Mrs., or Miss, etc.

All replies, addressed "Charity Box," THE HOSPITAL, Norfolk House, Norfolk Street, W.C., must arrive by Friday, Dec. 24th. Results will appear Saturday, Jan 1st, 1887.

*Every Competitor is required to cut out and enclose with his list the "Charity Box" Coupon* (see advertisement pages), *and a shilling entrance fee* (postal orders only, *crossed Lloyd's, etc., Banking Company, Limited*, THE HOSPITAL *Account.*)

The proceeds resulting from each "Charity Box" competition will be funded until the end of December 1886. The total sum available for distribution, and the names of the hospitals selected for donations, will appear on Saturday, January 8, 1887. The distribution of the proceeds will be at the Editor's sole discretion.

RESULTS OF TENTH COMPETITION

The "Medicine" Competition has resulted as follows:—

| | Accepted Score. |
|---|---|
| Mr. F. A. Robinson, Melrose House, Kingsley Road, Northampton .. .. .. .. .. } Mr. H. Klingemann, c.o. Messrs. E. Stavenhagen, 18, King's Arms Yard, Moorgate St., E.C. .. .. .. .. .. .. .. .. } | 49—1st and 2nd prize a "tie." |
| Mr. D. Cherrett, Newtown, Parkstone, Dorset | 48—3rd prize. |

The prizes have been forwarded to the winners.

**Special Notice.**

In the interest of non-successful competitors, we have decided not to award the First Prize more than once to a winner in any one quarter. In the event of a competitor again making the highest accepted score, he will be placed in the second place, and, if a third time highest, in the third place.

We must particularly ask correspondents not to swell their lists by the inclusion of purely Latin words, and abbreviations such as I'd, I'm, etc. Some competitors made over 60 words, and one over 70, from "Medicine"!

At Miss Williams's suggestion, half of the first prize last week has been placed to the credit of the "Charity Box" Account.

### NOTICE TO CORRESPONDENTS.

All correspondence must be written on one side of the paper only.

We cannot undertake to return MSS. not used.

**Communications,** whether intended for insertion or for private information, must be authenticated by the names and addresses of the writers, not necessarily for publication.

**Local papers** containing reports or news paragraphs **should be marked.**

It is especially requested that early intelligence of **local events of interest,** or which it may be thought desirable to bring under notice, may be sent direct to the Editor.

Communications respecting editorial matters should be addressed to the Editor, Norfolk House, Norfolk Street, Strand, London, W.C.

### BUSINESS COMMUNICATIONS AND ADVERTISEMENTS.

All communications concerning business matters, non-delivery of THE HOSPITAL, etc., should be addressed to the Manager, at the Publishing Offices, 30 and 32, Sardinia Street, Lincoln's Inn Fields, London, W.C.

**Cheap Advertisements** of the "Wanted" class, including those for situations, of Twenty-four Words or under, are charged 1*s.* an insertion, or three insertions for 2*s.* 6*d.*, but must be paid for in advance. Each additional line of Eight Words is 4*d.*

**Employers requiring assistants** are charged for one insertion of Twenty-four Words or under, 2*s.* 6*d.*, or 6*s.* for three insertions. Each additional line of Eight Words is 1*s.*

**Advertisements** for insertion in the weekly issue of THE HOSPITAL **must be delivered** at the Publishing Offices **not later than 11 a.m. on Wednesdays.**

### TERMS OF SUBSCRIPTION.

Subscribers to THE HOSPITAL residing in London and the Suburbs will receive their Copies by the first post on FRIDAY MORNING. Copies for Country Subscribers are forwarded by the DAY Mail on Fridays. Terms, including postage, payable in advance:

| | Weekly Parts. | Monthly Parts. |
|---|---|---|
| Twelve months | 0 6 6 | 0 7 6 |
| Six ,, | 0 3 3 | 0 3 9 |
| Three ,, | 0 1 8 | 0 1 11 |

Post Office Orders should be made payable at the General Post Office, London, E.C., to WHITING AND Co., 30 and 32, Sardinia Street, Lincoln's Inn Fields, W.C. Cheques to be crossed "London and Joint Stock Bank, Limited," Chancery Lane Branch.

**Cases for Binding** the weekly or monthly parts of THE HOSPITAL in yearly vols. may be ordered of the Publishers.

### The In- and Out-patients' Hospital Diary.

This Diary is so arranged as to make some portion of it appear every week, and the whole in each monthly part. It has been very difficult to compile, and corrections will at all times be gratefully received. It has been suggested that similar information about the larger Provincial and Scotch Hospitals would be useful to many readers. We should be glad to hear if this is felt to be the case.

### ORTHOPÆDIC CASES.

| Hospitals and Terms of Admission. | Physicians, Surgeons, and Medical Officers, with Days and Hours of Attendance. |
|---|---|
| City Orthopædic Hospital, 27, Hatton Garden, E.C. Sec., Mr. E. Dereuth. Admission free | *Surgeons*, Mr. E. J. Chance and House-Surgeon. M. Tu. Th. F. 2.30. New cases Tu. F. 2.30 |
| National Orthopædic Hospital for the Deformed, 234, Gt. Portland St, W. Sec., Mr. H. H. Canning. Free to poor; others by letter or payment | *In-Physician*, Dr. C. Beevor. *In- and Out-Surgeons*, Messrs. F. R. Fisher, M. Th. 2; W. J. Roeckel, Tu. F. 2; E. M. Little, W. 1 |
| Royal Orthopædic Hospital, 297, Oxford Street, W. Sec., Mr. B. Maskell. By Governor's letter | *Surgeons*, Messrs. B. E. Brodhurst, H. A. Reeves, C. Read. W. E. Balkwill, H. F. Baker. Daily, 1 |
| St. Bartholomew's Hospital, Smithfield, E.C. | *Surgeon*, Mr. Walsham, M. 2.30 |
| St. George's Hospital, Hyde Park Corner, S.W. | W. 2. *See* General Hospitals |

### PARALYSIS, EPILEPSY, AND DISEASES OF THE NERVOUS SYSTEM GENERALLY.

| | |
|---|---|
| Hospital for Diseases of the Nervous System, 32, Portland Terrace, Regent's Park Road, N.W. Sec., Mr. Howgrave Graham. By contributor's letter, or payment according to means, but free to necessitous poor | *In-Physicians*, Drs. J. Althaus, Th.; Hughes Bennett, M.; G. Ogilvie, Tu. 2. *Out-Physicians*, Drs. Laycock, F.; F. Hawkins, W.; J. Althaus, Th.; G. Ogilvie, Tu.; H. Bennett, M. Hour, 2. *In- and Out-Surgeons*, Messrs. J. Bloxam, A. P. Gould, M. Tu. W. Th. F. 2 |
| National Hospital for the Paralysed and Epileptic, Queen Square, Bloomsbury, W.C. Sec. and Director, Mr. B. B. Rawlings. A portion of the beds are reserved free to the necessitous poor; the others are for paying patients | *In-Physicians*, Drs. J. S. Ramskill, Tu.; C. B. Radcliffe, M.; J. H. Jackson, F.; T. Buzzard, W. Hour, 2.30. *In-Surgeons*, Messrs. W. Adams, M. 3; V. Horsley, F. 2.30. *Out-Physicians*, Drs. T. Buzzard, W., H. C. Bastian, Tu.; W. R. Gowers. M.; D. Ferrier, F. 2.30; J. A. Ormerod, Tu. W.; C. E. Beevor, M; F. Hour, 2.30 |
| National Hospital for Diseases of the Heart and Paralysis, 32, Soho Sq., W. Sec., Capt. F. Handley. In-patients require recommendation of Life Governor. | *In-Physicians*, Drs. V. Ambler and A. Duncan, M. W. Th. *In-Surgeon*, Mr. G. Bennett, Tu. *Out-Physicians*, Drs. V. Ambler, A. Duncan, R. Varley, J. Murray, M. 2, 7; Tu. 3; W 2, 8; Th. 2; F. 3. *Out-Surgeon*, Mr. G. Bennett, Tu. 5 |
| West End Hospital for Diseases of the Nervous System, 73, Welbeck St., W. Hon. Sec., Mr. H A. Dowell. Children only as in-patients. Out-patients by letter or payment. | *In- and Out-Physicians*, Drs. H. Tibbits, M. Th. 1.30; Stretch-Dowse, Tu. W. 1.30; S. H. Armitage, W. Tu. F. 6. *In- and Out-Surgeon*, Mr. Benson |

### DISEASES OF THE SKIN.

| | |
|---|---|
| Charing Cross Hospital, West Strand, W.C. | *Physician*, Dr. Sangster, M. 2 |
| Guy's Hospital, St. Thomas Street, S.E. | *Physicians*, Drs. Pye-Smith, Hale-White, Tu. 12 |
| Hospital for Diseases of the Skin, 52, Stamford St., S.E. Sec., Mr. S. Hayman. Free to the necessitous; also by payment | *Physician*, Dr. F. J. Payne. *Surgeons*, Messrs. J. Hutchinson, Waren Tay, and Wyndham Cottle. (Out-patients seen M. W. 3 Tu. Th. F. 2) |
| King's College Hospital, Portugal Street, W.C. | *Physician*, Dr. Duffin, F. 1 |
| London Hospital, Whitechapel Road, E. | *Physician*, Dr. S. Mackenzie, Th. 9 |
| Middlesex Hospital, Berners Street, W. | *Physician*, Dr. R. Liveing, Tu. 4 |
| North-West London Hosp. 18, Kentish Town Rd., N.W. | *Physician*, Dr. J. Stowers, Tu. 1.30 |
| Paddington Green Children's Hospital, Paddington | *Physician*, Dr. T. Calcott Fox, S. 9.15 |
| St Bartholomew's Hospital, Smithfield, E.C. | *Surgeon*, Mr. H. Cripps, F. 1.30 |

### DISEASES OF THE SKIN.—*Continued.*

| Hospitals and Terms of Admission. | Physicians, Surgeons, and Medical Officers, with Days and Hours of Attendance. |
|---|---|
| St. George's Hospital. Hyde Park Corner, S.W. | *Physician*, Dr. Cavafy, W. 1.30 |
| St. John's Hospital for Diseases of the Skin, 45, Leicester Sq., W.C. Branch, Markham Square, Chelsea, S.W. Sec., Mr. St. Vincent Mercier | *Physicians*, Drs. Robinson, W. 2, F. 7; Dow, Th. 2, M. 7; Harries, M. 2, W. 7. *Surgeon*, Mr. J. Milton, M. Tu. Th. F. 2 |
| St. Mary's Hospital, Cambridge Place, Paddington, W. | *Surgeon*, Mr. Morris, M. Th. 9.30 |
| St. Thomas's Hospital, Westminster Bridge Road, S.E. | *Physician*, Dr. Payne, W. 1.30 |
| University College Hospital, Gower Street, W.C. | *Physician*, Dr. Crocker, W. 1.30, S. 9.15 |
| Westminster Hospital, Broad Sanctuary, S.W. | *Physician*, Dr. T. C. Fox, W. 1.30 |

### STONE AND OTHER DISEASES OF URINARY ORGANS.

| | |
|---|---|
| London Hospital, Whitechapel Road, E. | Daily, 1 30. *See* General Hospitals |
| St. Peter's Hospital for Stone, Henrietta Street, W.C. Sec., Mr. W. E. Scott. Admission free. Only women and children are seen on Fridays. Operation day, Wednesday | *In-Surgeons*, Messrs. W. J. Coulson, F. R. Heycock. *Out-Surgeons*, Messrs. E. H. Fenwick, M. 2, S. 4 to 7; F. S. Edwards, M. 5 to 7, Th. 2; Heycock, Tu. 2, W. 5 to 7, F. 2 |

### DISEASES AND IRREGULARITIES OF THE TEETH.

| | |
|---|---|
| Belgrave Hosp. for Children, 77 & 79 Gloucester St., Pimlico | *Surgeon*, Mr. G. W. Payne, W. 9 |
| Charing Cross Hospital, West Strand, W C. | *Surgeon*, Mr. Fairbank, M. W. F. 9.0 |
| Dental Hospital of London, Leicester Sq., W.C. Sec., Mr. J. F. Pink. Poor free. For special operation a Governor's letter is required | *Surgeons*, Messrs. Canton, Gregson, Hepburn, S. Bennett, Underwood, Woodhouse, Hern, Matheson, Parkinson, Read, Rogers, Truman. Daily, 9 to 11 |
| Evelina Hosp. for Sick Children, Southwark Brdg. Rd. | *Surgeon*, Mr. R. Denison Pedley, Tu. 9 |
| German Hospital, Dalston lane, Dalston, E. | *Surgeons*, Messrs. A. P. Reboul, C. West, Th. 10 to 11 |
| Great Northern Central Hospital, Caledonian Rd, N. | *Surgeon*, Mr. E. Keen, W. 1.30 |
| Guy's Hospital, St. Thomas Street, S.E. | *Surgeons*, Messrs. Moon, Pedley, Tu. Th. F. 12.30 |
| Hospital for Consumption, Brompton, S.W. | *Surgeon*, Mr. C. J. Noble, W., and when wanted |
| Hospital for Sick Children, Gt. Ormond Street, W.C. | *Surgeon*, Mr. Cartwright, W. 9 |
| King's College Hospital, Portugal Street, W.C. | *Surgeon*, Mr. Cartwright, Tu. F. 10 |
| London Hospital, Whitechapel Road, E. | *Surgeon*, Mr. A. W. Barrett, Tu. 9 |
| London Temperance Hosp., Hampstead Road, N.W. | *Surgeon*, Mr. Adolphus Alexander, M. 2 |
| Metropolitan Free Hospital, Gray's Inn Road, W.C. | *Surgeon*, Mr. G. Curnock, Tu. Th. S. 9 |
| Middlesex Hospital, Berners Street, W. | *Surgeons*, Messrs. Bennett, M. 9.30; W. 9; Hern, W. 9, F. 9.30 |
| National Dental Hospital, 149, Gt. Portland Street, W. Sec., Mr. A. G. Klugh. The poor, and all urgent cases admitted free; others by Subscriber's letter | *Surgeons*, Messrs. H. Weiss, W. Weiss, M.; A. Smith, G. Bradshaw, Tu.; G. A. Williams, M. Davis, W.; A. F. Canton, H. G. Read, Th.; T. Gaddes, W. S. Thomson, F.; H. Rose, W. R. Humby, S. Hours 9 to 11 |
| National Orthopædic Hospital, Gt. Portland st, W. | *Surgeon*, Mr. H. Harris |

